# Monthly dynamics of microbial communities and variation of nitrogen-cycling genes in an industrial-scale expanded granular sludge bed reactor

**DOI:** 10.3389/fmicb.2023.1125709

**Published:** 2023-02-16

**Authors:** Kun Zhang, Yanling Zhang, Maocheng Deng, Pengcheng Wang, Xiu Yue, Pandeng Wang, Wenjun Li

**Affiliations:** ^1^School of Eco-environment Technology, Guangdong Industry Polytechnic, Guangzhou, China; ^2^School of Mechanics and Construction Engineering, Jinan University, Guangzhou, China; ^3^School of Food and Bioengineering, Guangdong Industry Polytechnic, Guangzhou, China; ^4^School of Environmental Science and Engineering, Sun Yat-sen University, Guangzhou, China; ^5^China National Electric Apparatus Research Institute Co., Ltd., Guangzhou, China; ^6^State Key Laboratory of Biocontrol and Guangdong Provincial Key Laboratory of Plant Resources, School of Life Sciences, Sun Yat-Sen University, Guangzhou, China

**Keywords:** EGSB, anaerobic activated sludge, microbial communities, physicochemical properties, nitrogen cycling, binning, viral communities

## Abstract

**Introduction:**

The expanded granular sludge bed (EGSB) is a major form of anaerobic digestion system during wastewater treatment. Yet, the dynamics of microbial and viral communities and members functioning in nitrogen cycling along with monthly changing physicochemical properties have not been well elucidated.

**Methods:**

Here, by collecting the anaerobic activated sludge samples from a continuously operating industrial-scale EGSB reactor, we conducted 16S rRNA gene amplicon sequencing and metagenome sequencing to reveal the microbial community structure and variation with the ever-changing physicochemical properties along within a year.

**Results:**

We observed a clear monthly variation of microbial community structures, while COD, the ratio of volatile suspended solids (VSS) to total suspended solids (TSS) (VSS/TSS ratio), and temperature were predominant factors in shaping community dissimilarities examined by generalized boosted regression modeling (GBM) analysis. Meanwhile, a significant correlation was found between the changing physicochemical properties and microbial communities (*p* <0.05). The alpha diversity (Chao1 and Shannon) was significantly higher (*p* <0.05) in both winter (December, January, and February) and autumn (September, October, and November) with higher organic loading rate (OLR), higher VSS/TSS ratio, and lower temperature, resulting higher biogas production and nutrition removal efficiency. Further, 18 key genes covering nitrate reduction, denitrification, nitrification, and nitrogen fixation pathways were discovered, the total abundance of which was significantly associated with the changing environmental factors (*p* <0.05). Among these pathways, the dissimilatory nitrate reduction to ammonia (DNRA) and denitrification had the higher abundance contributed by the top highly abundant genes *narGH*, *nrfABCDH*, and *hcp*. The COD, OLR, and temperature were primary factors in affecting DNRA and denitrification by GBM evaluation. Moreover, by metagenome binning, we found the DNRA populations mainly belonged to Proteobacteria, Planctomycetota, and Nitrospirae, while the denitrifying bacteria with complete denitrification performance were all Proteobacteria. Besides, we detected 3,360 non-redundant viral sequences with great novelty, in which *Siphoviridae*, *Podoviridae*, and *Myoviridae* were dominant viral families. Interestingly, viral communities likewise depicted clear monthly variation and had significant associations with the recovered populations (*p* <0.05).

**Discussion:**

Our work highlights the monthly variation of microbial and viral communities during the continuous operation of EGSB affected by the predominant changing COD, OLR, and temperature, while DNRA and denitrification pathways dominated in this anaerobic system. The results also provide a theoretical basis for the optimization of the engineered system.

## 1. Introduction

The bioavailable forms (e.g., ammonium and nitrate) of nitrogen is rare in natural environments, however, the rapid growth and development of human society make wastewater the largest possible source of nutrient (e.g., reactive nitrogen) in the environment, resulting in the eutrophication problems that could be experienced by receiving water bodies and ecosystems (Kuypers et al., 2018). Hence, reactive nitrogen treatment in wastewater is considered of key importance. The expanded granular sludge bed (EGSB) reactor is a developed anaerobic granular sludge technology involving the breakdown of biomass by a wide range of microorganisms in the anaerobic condition (Abdelgadir et al., 2014), which is a promising method for the treatment of various organic pollutants due to its energy effectiveness, limited nutrients requirements, and low sludge production (Xu et al., 2018). However, the performance of EGSB was reported to be strongly affected by the environmental parameters including high loading COD concentration and temperature (Cruz-Salomón et al., 2019). It is key important to monitor the changing physicochemical properties of the anaerobic sludge inside of the reactor in the long-term operation of an industrial-scale EGSB.

The microbial community in anaerobic sludge underpins wastewater treatment processes, from organic matter degradation and bioenergy generation to the removal of contaminants and recovery of nutrients such as nitrogen and phosphorus (Nielsen, 2017). Previous studies pointed out that the biological nitrogen removal-related pathways could be mainly divided into several pathways: nitrate reduction, denitrification, nitrification, anaerobic ammonium oxidation (Anammox), denitrifying anaerobic methane oxidation (DAMO), and ammonia assimilation (Welte et al., 2016; Holmes et al., 2019; Yang et al., 2020). The microbial taxa, which transform nitrogen compounds could be classified as nitrifiers, denitrifiers, diazotrophs, anaerobic ammonium oxidation (Anammox) microbiota, ammonia-oxidizing microbiota, or DAMO microbiota (Holmes et al., 2019; Madeira and de Araújo, 2021). For example, *Nitrosomonas*, *Nitrosospira*, and *Nitrosococcus* are reported as autotrophic ammonia-oxidizing bacteria (Anjali and Sabumon, 2022), while *Nitrobacter*, *Nitrospina*, and *Nitrotoga* are capable of chemolithoautotrophic nitrite oxidation (Daims et al., 2016). The composition and variation of the functional groups are thought to contribute to promoting process stability, sludge settling, and nutrient removal (Chen et al., 2017). Hence, research of the species and functional dynamics of microbial communities in anaerobic sludge underlying continuous operation of an EGSB contributes to a deep understanding of the spontaneously gathering mechanisms, which further guide the optimization of the engineered systems by deciphering the functional members and mainly metabolism pathways that appeared in it.

Since the advances in deep metagenomic sequencing and bioinformatics, the recovery of near-complete metagenome-assembled genomes (MAGs) directly from *in situ* environment has shed light on the myriad of important functions of microbiota. For instance, by direct inoculation of the exogenous anammox pellets and metagenomics binning, Yang et al. identified the anammox bacteria and reconstructed the overall microbial nitrogen-cycling networks in aeration tanks and deep oxidation ditches (Yang et al., 2020). Moreover, Meng et al. recently recovered multiple *Candidatus Brocadia* species in a full-scale swine wastewater treatment system and deciphered their co-occurring mechanisms in nitrogen metabolism (Meng et al., 2023). As known, engineered systems harbor diverse microenvironments with ever-changing chemical composition, exerting strong effects on population enrichment and co-occurrence. Previous research reported that along with the successive operation of wastewater treatment reactors, the microbial communities might occur a population shift due to niche overlap or nutrient consumption (Sun et al., 2021). Nevertheless, the research about the dynamics of functional members that participated in nitrogen cycling along with the long-term operation of an EGSB is scarcely limited.

Furthermore, studies suggested that the high biomass and sufficient nutrient in activated sludge formed a suitable habitat for viruses, in which the number of viruses might be 10 to a 1,000 times that in a natural aquatic ecosystem (Otawa et al., 2007). Recent research pointed out that viruses were widely distributed in wastewater treatment plants, which could lyse microbial cells or reprogram host metabolism exerting unknown but steady influences on the wastewater treatment processes (Li et al., 2021). However, research about the viral variation and the linkage with the host during the continuous operation of an EGSB is much less explored.

Here, 16S rRNA gene amplicon sequencing, high-throughput metagenome sequencing, and bioinformatics analysis were applied for anaerobic sludge collected monthly. We aimed to (i) reveal the composition and monthly variation of microbial communities, and viruses, (ii) investigate the nitrogen-related functional potentials and pathways, and (iii) elucidate the co-occurring mechanisms of different populations and physicochemical properties during a whole year of continuous operation of an industrial-scale EGSB. By monitoring the composition, function, variation, and interaction of microbial communities in the EGSB, we expanded our knowledge of the dynamic change of key members and nitrogen-related genes in this anaerobic system.

## 2. Materials and methods

### 2.1. Sampling site description and sample collection

An industrial-scale wastewater treatment plant is located in Guangdong Province, China (113°34′E, 22°56′N), which is designed to treating with municipal and industrial wastewater. The expanded granular sludge bed (EGSB) is set as the reactor of anaerobic active sludge and is operated at room temperature (Supplementary Figure S1), in which the sewage samples were collected from the upper, middle, and bottom layers of the EGSB at the end of each month from January to December 2020. All processes were repeated three times and finally, 108 samples were collected and immediately transferred to the laboratory on dry ice and stored at −40°C before the subsequent DNA extraction. Meanwhile, the influent wastewater from the hydrolysis acidification unit and the effluent wastewater from the EGSB were also collected each month for subsequent physiochemical analysis.

### 2.2. Physicochemical analysis

The pH and temperature were monitored monthly using a pH meter (Sartorius PB-10) and a thermometer separately. The different organic loading rates (OLRs) were achieved by various chemical oxygen demand (COD) concentrations at a fixed hydraulic retention time (HRT). The COD, total suspended solids (TSS), and volatile suspended solids (VSS) were measured according to standard methods (Rice et al., 2012). Biogas production was monthly monitored and the CH_4_ percentage was determined by Gas Chromatography (SRI 8610 C). The ammonium nitrogen and total nitrogen concentration were determined by the SmartChem Discrete Auto Analyzer (Smartchem200, AMS-Westco, Italy) and Shimadzu TOC Analyzer (TOC-Vcsh; Shimadzu), respectively.

### 2.3. DNA extraction, 16S rDNA, and metagenomics sequencing

The 108 genomic DNA were extracted from the sewage using the Qiagen PowerSoil DNA Kit (Qiagen) according to the manufacturer’s instructions. The extracted DNA was then transferred on dry ice to Guangdong Magigene Biotechnology Co., Ltd. (Guangzhou, China) for 16S rDNA and metagenomics sequencing. Briefly, the universal primer set 515F/806R was used for targeting the V4 region of 16S rDNA according to the method published previously (Zheng et al., 2022). The final amplicons were sequenced using a 2 × 250 bp paired-end method by the Illumina MiSeq platform. Other DNA samples for metagenomics were constructed libraries (with the insert size of 350 bp) and sequenced using a 2 × 150 bp paired-end method by the Illumina NovaSeq 6000 platforms. The amount of raw sequence data was ~12 Gb (40,828,360 paired-end sequences) per sample. The amplicon sequences and the metagenome sequences were deposited at NCBI Short Read Archive (SRA) under Bioproject accession No.: PRJNA896246.

### 2.4. Amplicon analysis, metagenome assembly, and annotation

The 16S raw paired-end reads were first conducted quality control by FASTP (Chen et al., 2018) and merged by FLASH v1.2.11 (Magoč and Salzberg, 2011). Then, clean sequences were analyzed according to the QIIME2 (Bolyen et al., 2019). Briefly, sequences were clustered into amplicon sequence variants (ASVs) of 100% similarity by q2-dada2.^1^ The ASVs table was rarified to the lowest sequence number (53,270) among all samples and the SILVA123 database was used for ASVs’ taxonomic assignment. The alpha diversity [Chao1, phylogenetic distance (Faith_PD), and Shannon indices] was then calculated for each sample. The Bray-Curtis distance-based PCoA was calculated for group dissimilarities.

The raw metagenome sequences were first conducted quality preprocessing by Trimmomatic (Bolger et al., 2014) for reads and adapter trimming with the following options: –Leading: 3 –Trailing: 3 –Slidingwindow: 4:15 –Minlength: 100. Then, the MegaHit (Li et al., 2015) was used for reads assembly with a k-mer of 21–99 in step of 6 and minimum contig length of 500 bp. Prodigal v2.6.3 (Hyatt et al., 2010) was used for gene prediction and a non-redundant gene set was constructed by CD-hit-est v4.6.6 (Fu et al., 2012) with parameters “–T 24 –M 0 –c 0.95 –G 0 –aS 0.9 –g 1 –d 0.” KOBAS 3.0 (Bu et al., 2021) was used for KEGG annotation (threshold−e 10–5).

### 2.5. Metagenome binning, annotation, and phylogenetic analysis

The MetaWrap pipeline (Uritskiy et al., 2018) invoking three independent packages (i.e., CONCOCT, metaBAT2, and MaxBin2) was applied for contigs binning, and MAGs de-replication was conducted according to the average nucleotide identity (ANI) > 95%, and the genome coverage >80%. Then, CheckM (Parks et al., 2015) was used to determine the genome quality, including the contamination, completeness, and strain heterogeneity of each obtained metagenome-assembled genome (MAG) based on the collocated sets of genes that are ubiquitous and single-copy within a phylogenetic lineage. The taxonomic identification of each MAG was evaluated by combining four independent methods including CheckM, GTDB-TK (Chaumeil et al., 2022), Taxator-TK (Dröge et al., 2015), and PhyloPhlAn 3.0 (Asnicar et al., 2020). ANI was calculated among MAGs and reference genomes downloaded from the NCBI RefSeq database using FastANI (Jain et al., 2018) by pairwise genomic comparisons, in which 95% ANI cutoff is the most frequently used standard for species demarcation and 70% ANI cutoff for the genus. The genome coverage is also set as an aligned fraction (AF) for assessment of similarity. The Prodigal was applied for gene prediction of individual genomes and KOBS 3.0 was used for KEGG annotation (threshold -e 10–5). KEGG Mapper (Kanehisa and Sato, 2020) was used for mapping the gene sets to the KEGG pathway and we calculated the presence or absence of genes involved in nitrogen pathways by the local script.

### 2.6. Viral sequences identification and host prediction

Vibrant v1.2.1 (Kieft et al., 2020) was used to detect and recover viral contigs longer than 5 kb. The complete circular viral contigs and the high-quality draft viral contigs were maintained. The predicted viral contigs were further examined by CheckV v0.8.1 (Nayfach et al., 2021) and removed the contigs set not determined. Cd-hit v4.7 (Fu et al., 2012) was applied to remove the redundancy of viral contigs (parameter: -c 0.95 -n 5 -g 1 -aS 0.8). Prodigal v2.6.3 (Hyatt et al., 2010) was used to predict viral genes. DRAMv v1.2.4 (Shaffer et al., 2020) was used for viral gene annotation. The virus-host linkage analysis was done following the two methods previously reported (Gao et al., 2022). Briefly, viral contigs were compared with the recovered MAGs using BLASTn (E-value ≤ 10–3, bit score ≥ 50, alignment length ≥ 2.5 kb, and identity ≥ 70%). The metaCRT (Rho et al., 2012) was applied for CRISPR spacers recovery from MAGs (parameters: default), while the spacers were compared to viral contigs by BLASTn (E-value ≤10–10 and no mismatches).

### 2.7. Statistical analysis

All statistical analyses were performed by R v4.0.3. Briefly, the phylogenetic tree of recovered population genomes was constructed by PhyloPhlAn 3.0 (Asnicar et al., 2020), and beautified by iTOL v5.5.1 (Letunic and Bork, 2021). The principle component analysis (PCA) and redundancy analysis (RDA) were analyzed using the vegan package. The significant difference (*p* < 0.05) among groups was analyzed using the Kruskal-Wallis test. The Procrustes analysis was conducted by the Vegan R package to compare the congruence between two different data sets based on the goodness of fit (M^2^) *p* value calculated by the Procrustes test and verified by the Mantel test. *p* < 0.05 was considered a significant difference.

## 3. Results

### 3.1. Performance of EGSB during continuous operation

**Figure 1:**
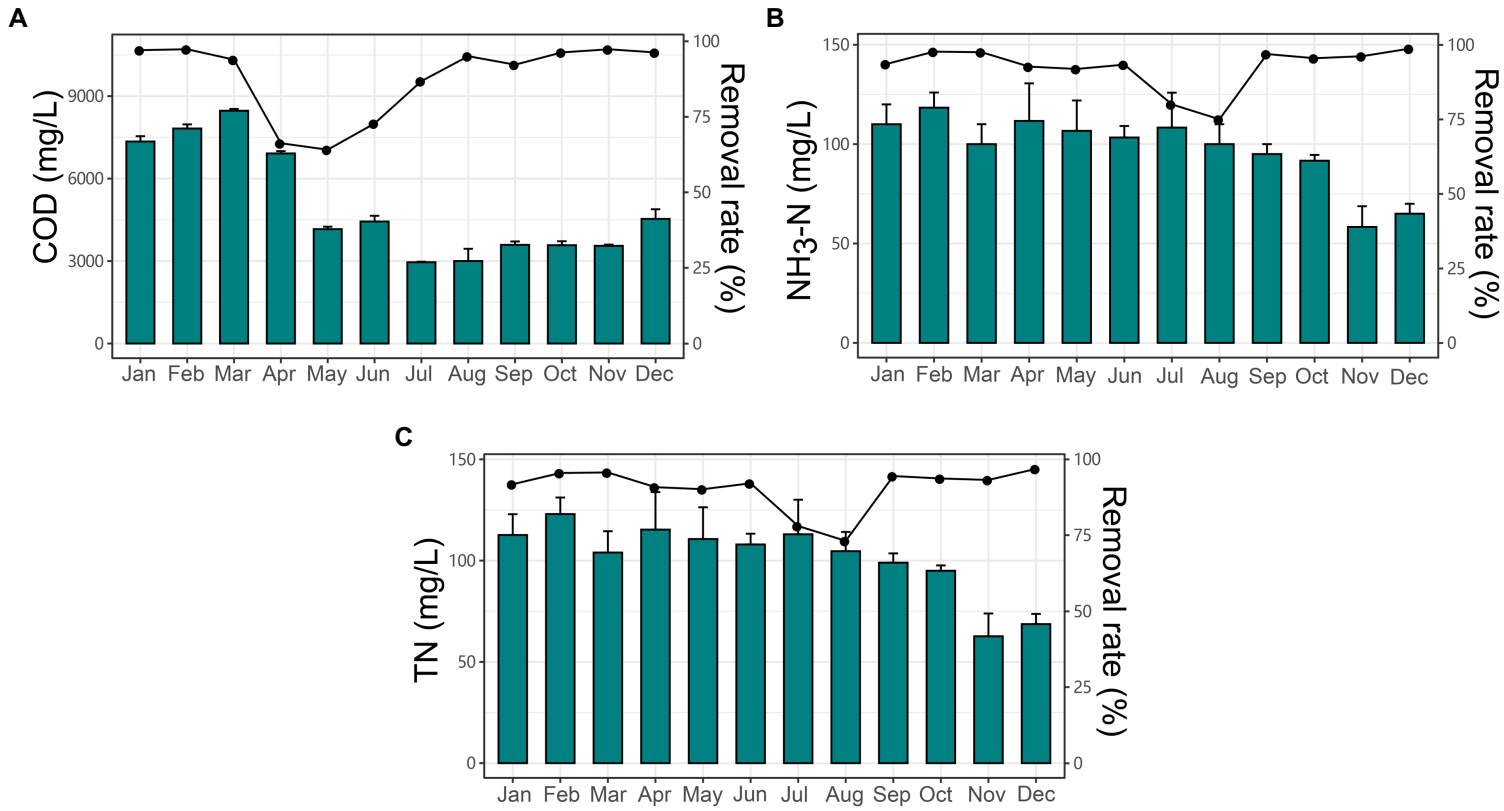
The changing trends and removal rate of nutrients in the expanded granular sludge bed (EGSB) reactor during 12 months. The concentration and removal rate (%) of **(A)** COD, **(B)** NH_3_-H, and **(C)** TN among different months.

The local industrial-scale EGSB reactor was applied for treating esterification wastewater. The EGSB was operated monthly with different organic loading rate (OLR) concentrations ranging from 17.31 to 48.16 kg COD/m^3^/d with the constant hydraulic retention time (HRT) of 64 h resulting in the biogas production ranging from 259.27 to 456.07 l/kg COD. The percentage of methane was ranging from 73 to 86% of total biogas. The COD removal efficiency was ranging from 64.11 to 97.34% (Figure 1A) with the maximum efficiency achieved in February at the OLR of 45.20 kg COD/m^3^/d. Besides, the inner temperature of the reactor was ranging from 8 to 39°C and the pH of the anaerobic activated slugged remained slightly alkaline (7.08 ± 0.14). The ratio of volatile suspended solids (VSS) to total suspended solids (TSS; VSS/TSS ratio) was ranging from 50.07 to 65.55%. Detailed information about the operation parameter could be found in Table 1. Specifically, the average TN of anaerobic sludge was ranging from 62.67 ± 9.18 to 123 ± 6.68, with a removal rate ranging from 73.2 to 96.9% (Figure 1B), while the average NH_3_-N was ranging from 58 ± 8.5 to 118 ± 6.24, with the removal rate ranging from 75 to 98.7% (Figure 1C).

**Table 1:** The detailed information about the operation parameter of EGSB.

Month	OLR ^a^ (kg COD/m3/d)	Biogas production ^b^ (l/kg COD)	CH_4_%	pH	Inner temperature (°C)	VSS/TSS ^c^ (%)
Jan.	40.64	456.07	79%	6.94	8°C	62.19%
Feb.	45.20	374.35	82%	7.21	10°C	61.15%
Mar.	48.16	320.28	83%	6.95	25°C	59.19%
Apr.	27.90	275.27	80%	7.4	32°C	56.79%
May.	17.31	297.79	77%	7.23	34°C	54.91%
Jun.	23.10	259.27	73%	7.11	35°C	50.07%
Jul.	21.66	265.92	82%	6.92	38°C	63.01%
Aug.	37.34	305.91	82%	6.95	39°C	61.43%
Sep.	34.62	378.35	84%	7.11	37°C	62.11%
Oct.	38.36	313.46	86%	7.18	30°C	61.61%
Nov.	31.11	388.47	85%	6.97	28°C	65.55%
Dec.	40.25	400.65	84%	7.01	20°C	61.51%

^a^Organic loading rate; ^b^a biogas production is given in liters per kg COD eliminated for EGSB; ^c^volatile suspended solids (VSS)/total suspended solids (TSS). The hydraulic retention time (HRT) and the solid retention time (SRT) were constant and fixed at 64 h and 350 days, respectively.

### 3.2. Monthly variation of microbial composition and diversity in anaerobic-activated sludge

**Figure 2:**
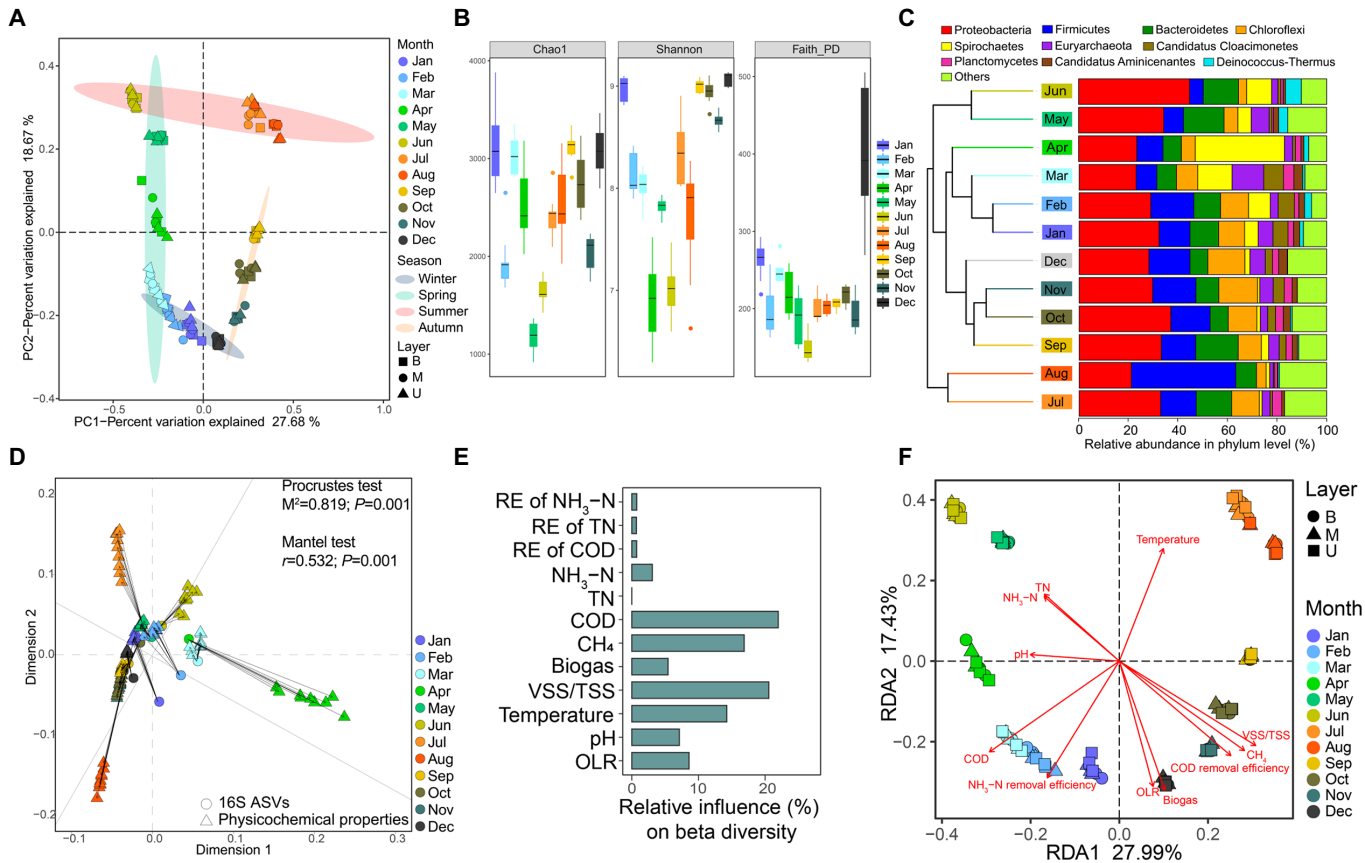
Overview of the composition, diversity, and variation of microbial communities with the changing physicochemical properties during the continuous operation of EGSB. **(A)** PCoA of bacterial communities in anaerobic activated sludge based on Bray–Curtis distances. Different color represents the sampling month and different symbol represents the sampling layer of the reactor. **(B)** Distribution of alpha indices (Chao1, Shannon, and Faith_PD) over 12 months. **(C)** Microbial community compositions at the phylum level across 12 months. Samples are collapsed and colored by month and clustered based on their Bray–Curtis distances. **(D)** Procrustes analysis of the changing physicochemical properties and the abundance of ASVs. Both the Procrustes test and the Mantel test are used for evaluating whether the association is significant and *p* < 0.05 is considered a significant correlation. **(E)** The relative contributions of different physicochemical properties to the group dissimilarities are determined by GBM analysis. **(F)** The ordination plot of redundancy analysis (RDA) for the microbial community. The relative variance explained by each axis was labeled in the axis title.

A total of 108 samples generated 17,149,173 high-quality amplicons with 158,789 per sample, and samples were rarified to the same sequencing depth (53,270) for normalization. By clustering based on 100% sequence similarity, 232,352 amplicon sequence variants (ASVs) were obtained and the average number of ASVs was 2151.41 ± 515.25 per sample. First, based on the Bray-Curtis distance PCoA constructed from the ASVs table, we observed that samples could be finely clustered according to the same sampling month, while different sampling layers exhibited little effects on group dissimilarities (Figure 2A). Surprisingly, we noticed the obvious seasonal variation among samples, indicating the potential effects of the ambient temperature on EGSB.

Then, we found that the species richness (Chao1 index) varied among months ranging from 1180.95 ± 147.78 to 3132.2 ± 382.2 with the highest value in January and the lowest in May. The whole species diversity (Shannon index) was ranging from 6.93 ± 0.43 to 9.06 ± 0.07 with the highest diversity in December and the lowest in April. The phylogenetic diversity (Faith_PD index) was ranging from 149.85 ± 18.67 to 428.23 ± 157.19 with the highest in December and the lowest in June (Figure 2B). Moreover, we examined the significant difference in alpha indices (Chao1, Shannon, and Faith_PD) among different seasons (Table 2). The result indicated that samples in winter owned the significantly highest species richness (Chao1) and the largest phylogenetic distances (Faith_PD), while the significantly lowest Chao1 and Faith_PD appeared in summer. The Shannon was significantly higher in autumn and winter compared with spring and summer. Besides, generalized boosted regression modeling (GBM) analysis indicated that the OLR was the major factor in regulating both Chao1 and Faith_PD, while VSS/TSS ratio showed the biggest influence on Shannon (Supplementary Figure S2).

**Table 2:** Calculation of significant differences of alpha indices among different seasons.

Alpha indices	Spring	Summer	Autumn	Winter
Chao1	2239.04 ± 837.89 bc	2193.47 ± 483.15 c	2635.55 ± 505.98 ab	2721.94 ± 629.18 a
Faith_PD	219.11 ± 36.57 b	184.06 ± 29.02 c	205.14 ± 18.03 b	296.52 ± 133.53 a
Shannon	7.59 ± 0.55 b	7.69 ± 0.67 b	8.87 ± 0.17 a	8.72 ± 0.45 a

Values are represented as mean ± SD (*n* = 27).

Different lowercase letters within the same row indicate significant differences among different seasons (Kruskal-Wallis, *p* < 0.05).

By taxonomic classification of ASVs, we found that Proteobacteria (24.84% ± 5.75%), Firmicutes (12.32% ± 7.77%), Bacteroidetes (8.85% ± 2.89%), Chloroflexi (7.35% ± 2.85%), Spirochaetes (6.31% ± 8.26%), and Euryarchaeota (3.97 ± 2.46%) were main abundant phyla within the anaerobic sludge samples (Figure 2C), while the successive variation of species composition was also observed along the year timeline. Interestingly, the abundance of Firmicutes was dramatically higher in August, while Spirochaetes occupied a larger proportion in April both compared with other months.

Further, the Procrustes analysis indicated that the alteration of changing physicochemical properties was significantly correlated with the variation of microbial communities (Figure 2D). Meanwhile, COD concentration, VSS/TSS ratio, CH_4_, and temperature explained over 70% of the total community variation by GBM analysis (Figure 2E). Moreover, by redundancy analysis (RDA), multiple factors showed different effects on sample variation (Figure 2F). For instance, temperature showed the most significant positive effects on samples in May, June, July, August, and September. COD and pH were significantly positively associated with samples in February, March, and April. The VSS/TSS ratio, CH_4_ percentage, and OLR showed significant positive correlations with samples from January, December, November, and October.

### 3.3. Composition and fluctuation of nitrogen-cycling genes

**Figure 3:**
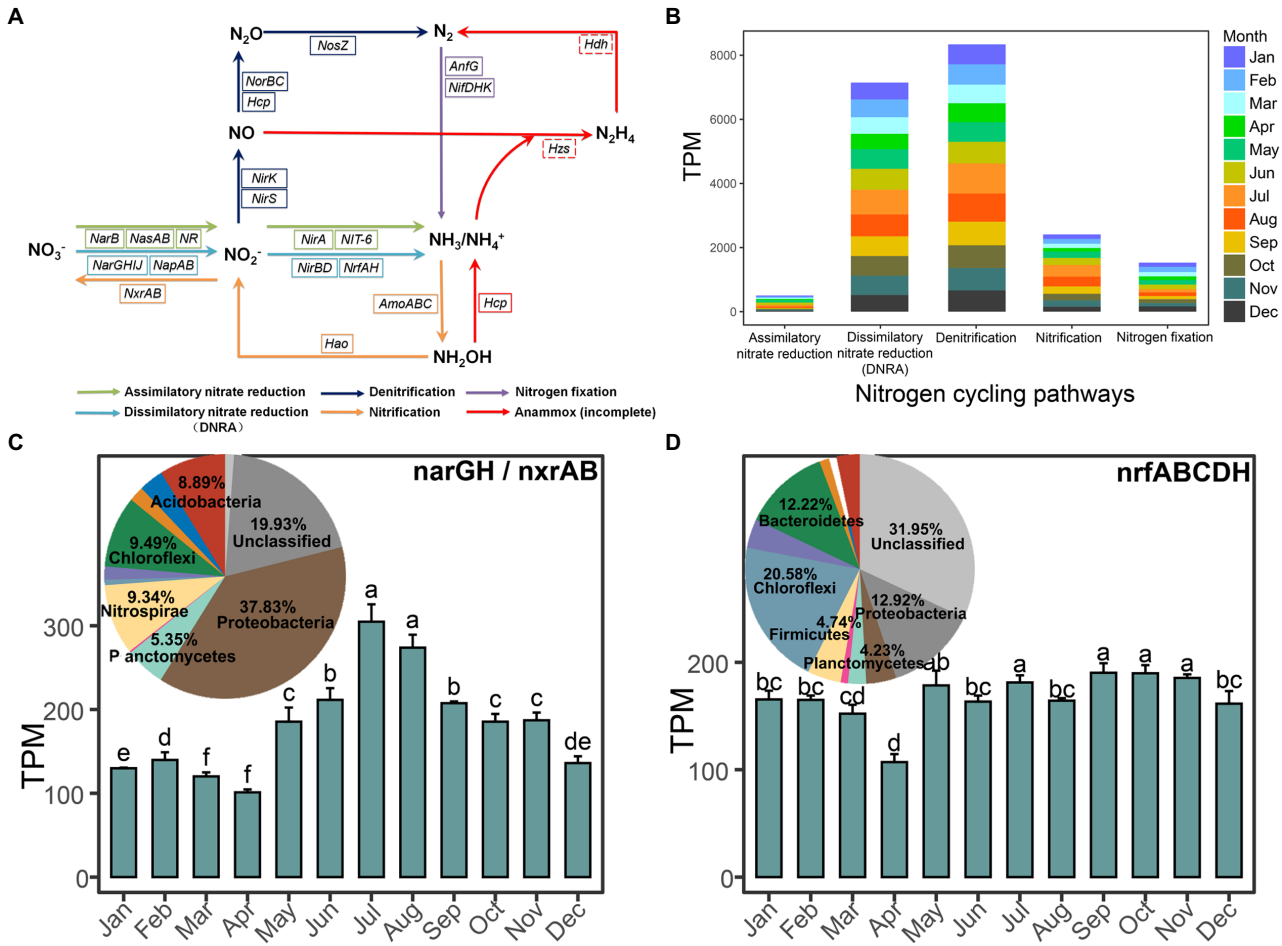
Overview of the key nitrogen-related genes and pathways in this anaerobic-activated sludge. **(A)** Key genes in the nitrogen cycling pathways. A solid box of genes represents the gene present and a dashed box of genes represents the gene absent in this EGSB system. **(B)** The stacked bar chart displays the proportions of the nitrogen cycling pathways represented by different months. Months are color-coded. **(C,D)** show the relative abundance of the most highly abundant genes *narGH*/*nxrAB* and *nrfABCDH* among each month, respectively, and their taxonomic assignment.

Metagenome sequencing of 36 activated sludge samples generated nearly 440 Gb raw data with 12 Gb data per sample. Through reads assembly, we obtained 11,790,544 contigs, resulting in 8,866,059 non-redundant genes, in which 3,439,726 genes could be successfully assigned to the KEGG database and 27,815 non-redundant genes could be annotated into 18 key types of genes covering nitrate reduction pathway, denitrification pathway, nitrification pathway, and nitrogen fixation pathway (Figure 3A). Unfortunately, no hits for the key hydrazine dehydrogenase gene (*hdh*) and hydrazine synthase gene (*hzs*) involved in the anammox pathway were searched, which indicated the possible loss of such biological nitrogen removal pathway in our dataset. We found that the dissimilatory nitrate reduction to ammonia (DNRA), denitrification, and nitrification pathways were the three most abundant pathways in our dataset by examining the total abundance of key genes involved in the complete pathways (Figure 3B).

Moreover, we calculated the abundance of each nitrogen-related gene and analyzed the contribution and composition of the main phyla, which the functional genes derived from. Among these genes, the nitrate reductase gene (*narGH*) or nitrite oxidoreductase gene (*nxrAB*) functioning in the transformation of nitrate with nitrite had the highest abundance (181.93 ± 59.21) in samples and was significantly highest in both July and August and significantly lowest in both March and April (all *p* < 0.05; Figure 3C). Approximately 37.83% of total *narGH*/*nxrAB* was assigned to Proteobacteria, followed by 9.46% to Chloroflexi and 9.34% to Nitrospirae. The second highly abundant nitrite reductase gene (*nrfABCDH*; 167.02 ± 21.62), functioning in the DNRA pathway, mainly came from Chloroflexi (20.58%), Proteobacteria (12.92%), and Bacteroidetes (12.22%; Figure 3D) and was significantly lowest in April (*p* < 0.05). Besides, the following abundant genes hydroxylamine reductase gene (*hcp*; 154.06 ± 28.89), nitrogenase molybdenum-iron gene (*nifDHK*; 126.41 ± 21.76), and nitrite reductase (NADH) gene (*nirBD*; 115.70 ± 18.94) showed capacity in ammonium formation or nitrogen reduction and all significantly higher in April (Supplementary Figure S3). Proteobacteria (43.44%) and Nitrospirae (13.64%) were mainly responsible for *nirBD*, while Euryarchaeota, Proteobacteria, and Firmicutes were the three predominant phyla that contributed to nitrogen fixation by *nifDHK* and hydroxylamine reduction to ammonium or nitric oxide reduction to nitrous oxide by *hcp*. Besides, the genes involved in the denitrification pathway including the nitrite reductase (NO-forming) gene (*nirK*/*nirS*; 75.17 ± 21.23), nitric oxide reductase gene (*norBC*; 100.01 ± 19.41), and nitrous-oxide reductase gene (*nosZ*; 52.79 ± 14.69) ranked top 10 abundant and were all significantly higher in July and August (Supplementary Figure S3). Proteobacteria (37.77%) and Chloroflexi (33.05%) accounted for over 70% abundance of *nirK*/*nirS* in regulating nitrite reduction to nitric oxide, while the compositions of main phyla were similar for *norBC* and *nosZ* that Proteobacteria and Bacteroidetes all contributed over 50% of the total abundance of them. Additionally, most of the genes participating in assimilatory nitrate reduction pathways were extremely low abundant (e.g., *NR*, *narB*, *nirA*, and *NIT-6*). Detailed information could be found in Table 3.

**Table 3:** Abundance of the nitrogen-related genes within 36 metagenomes.

Nitrogen-related genes	Abbreviation	Mean ± SD (TPM)
Nitrate reductase/Nitrite oxidoreductase	*narGH*/*NxrAB*	181.93 ± 59.21
Nitrite reductase (cytochrome c-552)	*nrfABCDH*	167.02 ± 21.62
Hydroxylamine reductase	*hcp*	154.06 ± 28.89
Nitrogenase molybdenum-iron protein	*nifDHK*	126.41 ± 21.76
Nitrite reductase (NADH)	*nirBD*	115.70 ± 18.94
Nitric oxide reductase	*norBC*	100.01 ± 19.41
Nitrate reductase molybdenum cofactor assembly chaperone	*narIJ*	85.79 ± 17.57
Nitrite reductase (NO-forming)	*nirK*/*nirS*	75.17 ± 21.23
Nitrous-oxide reductase	*nosZ*	52.79 ± 14.69
Nitrate reductase (cytochrome)	*napAB*	45.12 ± 10.30
Assimilatory nitrate reductase	*nasAB*	32.39 ± 12.07
Methane/ammonia monooxygenase	*amoABC*	13.20 ± 13.53
Ferredoxin-nitrite reductase	*nirA*	7.71 ± 5.53
Hydroxylamine dehydrogenase	*hao*	5.64 ± 3.16
Nitrogenase delta subunit	*AnfG*	1.10 ± 1.00
Nitrate reductase (NAD(P)H)	*NR*	0.96 ± 0.85
Ferredoxin-nitrate reductase	*narB*	0.66 ± 0.41
Nitrite reductase (NAD(P)H)	*NIT-6*	0.25 ± 0.17
Hydrazine dehydrogenase	*hdh*	0
Hydrazine synthase	*hzs*	0

Values are represented as mean ± SD (*n* = 36).

**Figure 4:**
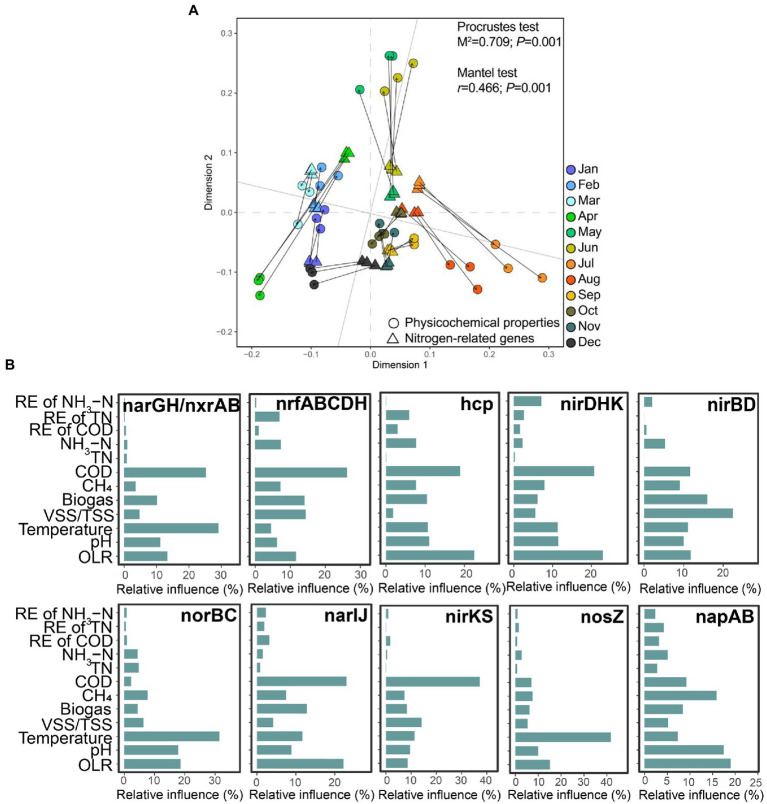
Correlation between the physicochemical properties and the nitrogen-related genes. **(A)** Procrustes analysis of the changing physicochemical properties and the abundance of nitrogen-related genes. Both the Procrustes test and the Mantel test are used for evaluating whether the association is significant and *p* < 0.05 is considered a significant correlation. **(B)** The relative contributions of different physicochemical properties to the top 10 highly abundant genes are determined by GBM analysis.

Importantly, we observed a significant correlation between the changing trends of the physicochemical properties and the total variation of nitrogen-related genes during the operation of this EGSB (Figure 4A). We further applied GBM analysis to reveal the relative influence of physicochemical properties on each highly abundant nitrogen-related gene. The result showed that seven environmental factors (COD, CH_4_, biogas production, VSS/TSS ratio, temperature, and OLR) mainly accounted for the variance of the top 10 abundance of nitrogen-related genes. Briefly, the concentration of COD exerted a predominant influence on *nrfABCDH*, and *nirK*/*nirS*, while OLR showed the biggest influence on *hcp*, *nirDHK*, and *napAB*. The temperature was the dominant factor on *narGH*, *norBC*, and *nosZ*.

### 3.4. Nitrogen-cycling-related populations

To better link the specific taxa and nitrogen function, we recovered 172 non-redundant and high-quality MAGs (MAG1 to MAG172) with completeness higher than 90% and contamination lower than 5%. The genome size varied from 1.08 to 8.21 Mbp among these MAGs with 3.52 ± 1.32 Mbp per MAG, while the GC content was 56.3% on average. Detailed information could be found in Supplementary Table S1. By phylogenetic placement, four MAGs could not be classified at the phylum level, while 168 MAGs could be successfully classified into 29 phyla (Supplementary Figure S4), mainly including Proteobacteria (31 MAGs), Bacteroidetes (26 MAGs), and Planctomycetes (20 MAGs). Specifically, 11 MAGs were annotated as Archaea. Moreover, the result of the ANI calculation by FastANI indicated that most of the MAGs showed a divergent phylogenetic distance from the known reference genomes (Supplementary Table S2), indicating that the majority of the MAGs recovered from the anaerobic-activated sludge seemed to be new with a high degree of novelty. By gene prediction and functional annotation, 171 MAGs contained one or more functional nitrogen-related genes (Supplementary Table S3), and multi-MAGs owned full genes in regulating the nitrate reduction to ammonium, denitrification, and nitrogen fixation pathways.

**Figure 5:**
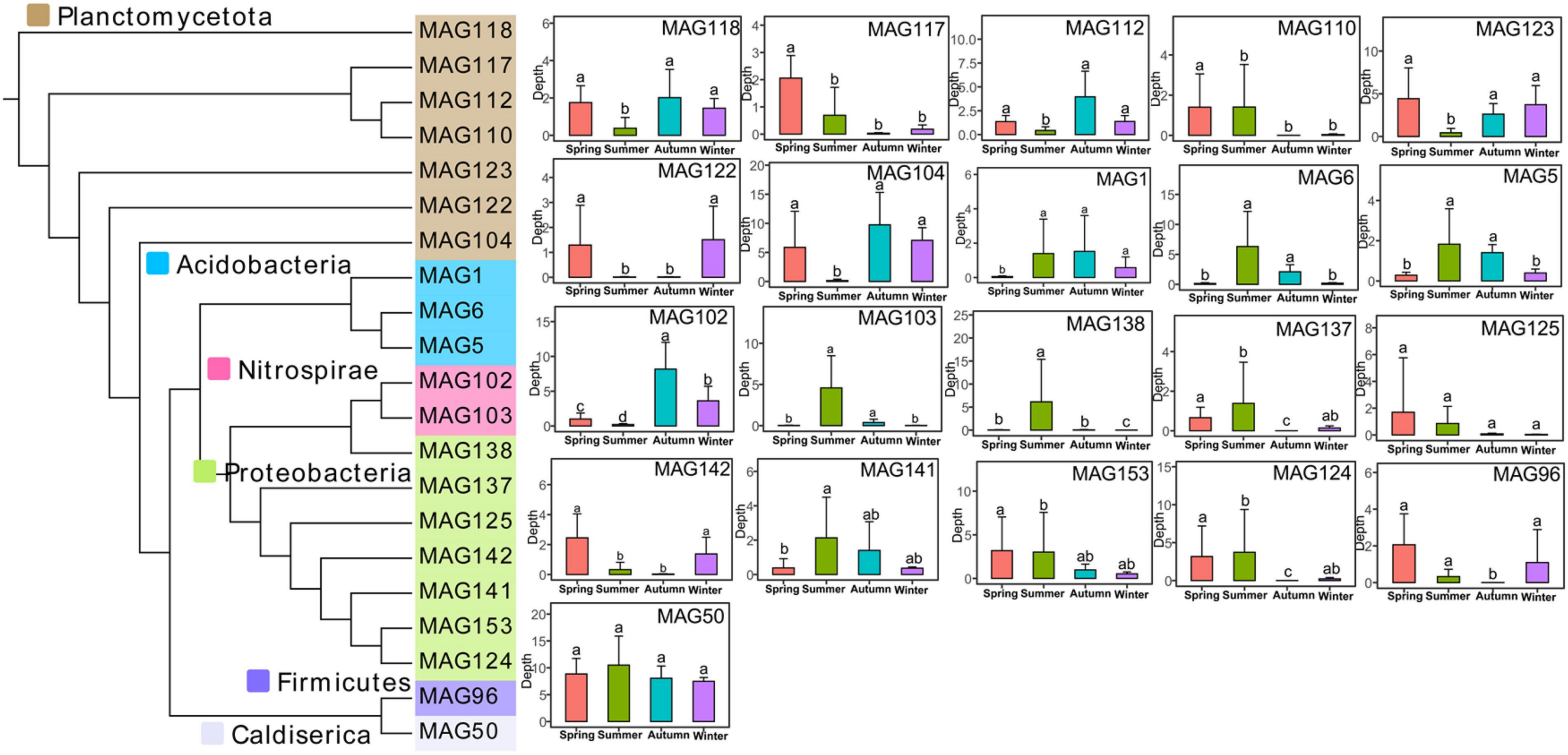
Phylogenetic tree of the recovered MAGs in nitrate reduction to ammonium and significant difference among the four seasons of each MAG. Different lowercase letters indicate significant differences among different seasons (Kruskal-Wallis test, *p* < 0.05).

Briefly, 24 MAGs owned *nasA* and 11 MAGs owned *nirA*, the two key genes in the assimilatory nitrate reduction pathway. For the DNRA pathway successively catalyzed by *narG* and *nirBD* or by *napA* and *nrfAH*, 26 MAGs had the *narG* and 48 MAGs owned *nirBD*, while 19 MAGs annotated the *napA* gene and 48 MAGs annotated the *nrf*. Five MAGs and 18 MAGs had full capacities in the assimilatory nitrate reduction and DNRA pathways, respectively. In keeping with the functional taxonomic assignment at the gene level, MAGs belonging to Planctomycetota, Proteobacteria, Acidobacteria, and Nitrospirae could conduct nitrate reduction directly to ammonium (Figure 5). Most of the MAGs belonging to Planctomycetota were significantly higher in spring (March, April, and May) and winter (December, January, and February), but dramatically lower in summer (June, July, and August). MAGs belonging to Acidobacteria significantly downregulated in the colder month (spring and winter) compared with the warmer month (summer and autumn). The phylum Nitrospirae MAG102 and MAG103 showed the highest abundance in autumn (September, October, and November). The phyla Proteobacteria contained seven MAGs, which varied disorderedly among four seasons, however, most of their abundance showed a decrease in the latter half of the year. The Firmicutes MAG96 (*Sporomusa* sp.) was significantly lower in autumn, while the Caldiserica MAG50 (*Cryosericum sp*.) showed no significant difference among the four seasons.

Five MAGs had full capacities in denitrification pathways by directly catalyzing nitrite to nitrogen, which all belonged to the phyla Proteobacteria (Supplementary Figure S5). They were complete denitrifiers, whereas they showed different trends among seasons. Briefly, MAG126 (*Pinisolibacter sp*.) and MAG147 (*Rhizobiales* bacterium) had a similar abundance pattern along with 12 months, while MAG149 (*Thauera sp*. K11) and MAG134 (*Rhodobacteraceae* bacterium) were much closer. Besides, among the 172 MAGs, 26 MAGs could conduct nitrogen fixation, including nine Proteobacteria, six Firmicutes, six Euryarchaeota, two thermoplasmatota, one Bacteroidetes, one Fusobacteria, and one Elusimicrobia. MAGs showed different patterns within a year and no obvious trends were found between MAGs belonging to the same phylum (Supplementary Figure S6).

Another essential process in nitrogen cycling in this dataset reflected by gene level was the nitrification pathway. Only MAG161 (unclassified *Thaumarchaeota*) owned *amo* gene to catalyze ammonia to hydroxylamine, while MAG132 (*Nitrosomonas nitrosa*) and MAG72 (*Elusimicrobia* bacterium) had the *hao* gene to catalyze the hydroxylamine to nitrite. A total of 27 MAGs (excluding MAG161, MAG132, and MAG72) could conduct nitrite oxidation with the *nxrAB* gene. However, none was identified as complete ammonia oxidizes (Comammox) owning all three key enzymes in this anaerobic active sludge.

### 3.5. Composition and variation of viral communities in anaerobic active sludge

**Figure 6:**
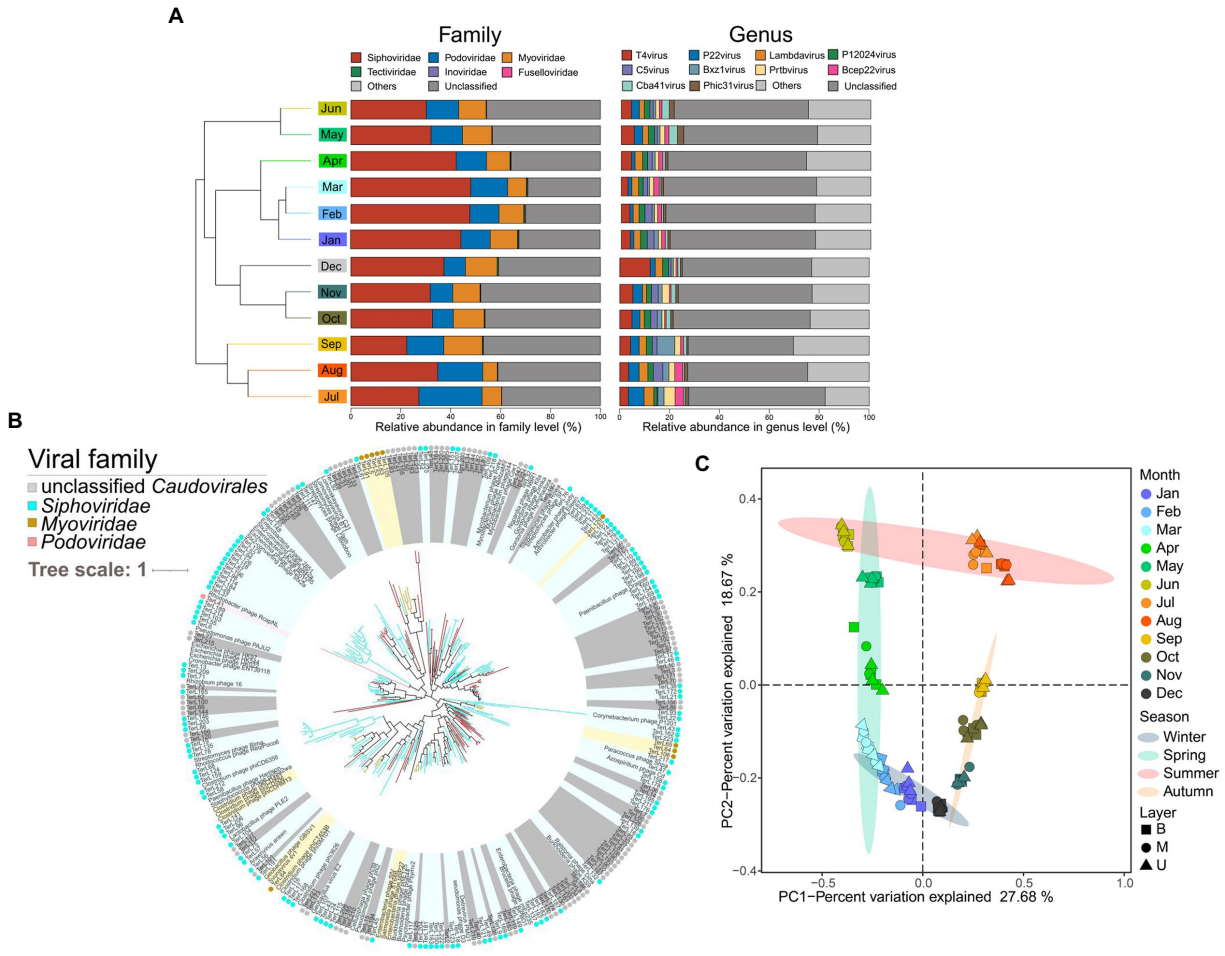
Overview of the viral communities in the EGSB. **(A)** Composition of the viral community-based at family and genus level. Samples are collapsed and colored by month and clustered based on their Bray–Curtis distances. **(B)** Phylogenetic tree based on the *terL* gene of *Caudovirales*. The Viral family-specific protein marker *terL* gene is used to construct phylogenetic trees with references downloaded from viral RefSeq for *Caudovirales*. Light yellow, light blue, light pink, and gray nodes and circles outside represent *Myoviridae*, *Siphoviridae*, *Podoviridae*, and unclassified *Caudovirales*, respectively. The branches of undetermined viruses are colored red. **(C)** PCoA of viral communities in anaerobic activated sludge based on Bray–Curtis distances. Different color represents the sampling month and different symbol represents the sampling layer of the reactor.

A total of 3,360 non-redundant putative viral sequences with lengths ranging from 5 to 505 kb were finally obtained in this dataset, while *Siphoviridae* (35.91 ± 7.84%), *Podoviridae* (13.35 ± 4.54%), and *Myoviridae* (10.44 ± 2.54%) were three main viral families all belonged to the order *Caudovirales* (Figure 6A). Specifically, we found that the viral family *Siphoviridae* was significantly higher in the colder months (spring and winter). The *Podoviridae* was significantly lower in autumn and winter, while the *Myoviridae* showed the opposite trend (Supplementary Figure S7).

Besides, *T4virus* (4.74 ± 2.41%), *P22virus* (2.94 ± 1.45%), *Lambdavirus* (2.63 ± 0.68%), and *P12024virus* (2.21 ± 0.40%) were the main classifiable viral genera (Figure 6A), whereas the majority of the viruses remained unknown. Protein sequences of 227 terminase large subunits (*terL*) were extracted from the viral set followed by constructing the phylogenetic tree (Figure 6B); indicating a great diversity and novelty of the viruses compared with the known reference proteins from NCBI RefSeq. Then, we examined the distribution patterns of viral populations among 36 samples by PCA. Interestingly, the result was consistent with the distribution trends of microbial communities. Briefly, samples from the same month shared similar viral compositions and were separated clearly along with the different seasons (Figure 6C), whereas no obvious trends in different layers of the tank were found.

### 3.6. Linkages between the viral contigs and the MAGs

**Figure 7:**
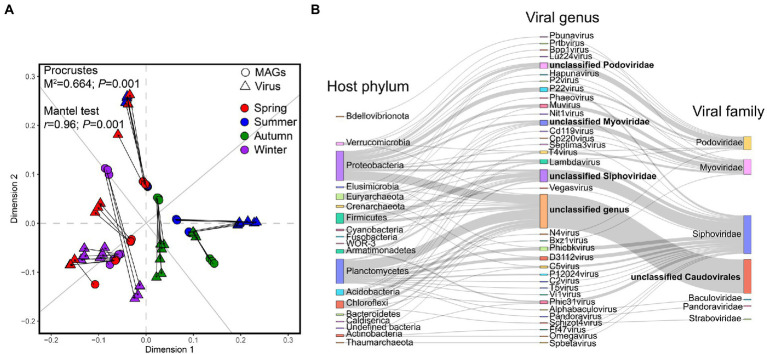
Correlation between the viruses and recovered MAGs. **(A)** Procrustes analysis shows the correlation between the abundance of viral community and the abundance of nitrogen-related genes. Both the Procrustes test and the Mantel test are used for evaluating whether the association is significant and *p* < 0.05 is considered a significant correlation. **(B)** Sankey diagram shows the linkages of viral sequences and the hosts based on CRISPR-Cas and genomic similarity blasting with MAGs.

The viral abundances showed a significantly positive correlation with the abundance of 172 MAGs within 36 samples determined both by Procrustes (*M*^2^ = 0.66, *p* = 0.001) and the Mantel test (*r* = 0.96, *p* = 0.001; Figure 7A), indicating that the coupling between the viral communities and the recovered MAGs in active sludge is significantly strong in different seasons throughout the year. To reveal the potential viral-host linkages, we detected the CRISPR-Cas spacers in host genomes, while 30 spacers were found in 12 MAGs (Supplementary Table S4). The 172 high-quality MAGs were also conducted for genomic alignment with viral sequences. As a result, 198 viral sequences were successfully linked to 72 MAGs forming 206 linkages, among which six viral sequences could match two different loci on the same host separately (Supplementary Table S5). The hosts spanned 19 phyla mainly including Proteobacteria (20 MAGs), Planctomycetes (15 MAGs), Firmicutes (seven MAGs), Chloroflexi (seven MAGs), and Acidobacteria (four MAGs), while the viruses contained 33 known different types of viral genera belonging to six known viral families with a big proportion of unclassified *Caudovirales* (Figure 7B).

Importantly, the predicted viral genes were annotated against the eggNOG database, in which the genes in categories C, E, F, G, H, I, and P were broadly considered auxiliary metabolic genes (AMGs; Chen et al., 2021). We filtered four viruses-owned genes that contributed to nitrogen metabolism. Briefly, U_ctg467926 (*Cd119virus*) had an *hcp* gene in reducing ammonium hydroxide to ammonium, which could interact with MAG89 (*Peptococcaceae* bacterium). U_ctg7461252 (unclassified *Myoviridae*) had a *nifU* gene related to nitrogen fixation and had a linkage with MAG68 (*Cyanobacteria* bacterium). U_ctg6130193 (unclassified *Caudovirales*) had an *npd* gene for nitronate monooxygenase and can interact with MAG134 (*Rhodobacteraceae* bacterium). U_ctg1124074 (unclassified *Caudovirales*) had an uncharacterized protein involved in response to NO and can interact with MAG126 (*Pinisolibacter* sp.).

## 4. Discussion

Anaerobic activated sludge system, a unique artificial microbial ecosystem with abundant diversity and high biomass concentration, efficiently aggregates the functional microbial groups to guarantee stable and good performance of biological wastewater treatment, in which the functional groups play key roles in the ecological biogeochemical cycle and elements energy flow (Saunders et al., 2016; Zhang and Zhang, 2022). The expanded granular sludge bed (EGSB) reactor has been widely applied in wastewater treatment, which is deemed to be low energy consumption and a type of clean energy producer (Castrillón Cano et al., 2020). In the present study, an industrial-scale EGSB reactor was set at room temperature and continuously running for a year dealing with the esterification wastewater, which generated methane-rich biogas monthly (Table 1).

By 16S rRNA gene sequencing, we observed a clear monthly and seasonal variation of microbial communities (Figure 2A). In line with our result, previous studies suggested that ambient temperature was an important driver for microbial diversity patterns including population synchrony and shifts in broad phylogenetic abundance (Griffin and Wells 2017), and revealed the seasonal dynamics of the microbial communities in activated sludge, especially between the warmer and colder months (Cai et al., 2020; Sun et al., 2021). Although, the sludge flocs contain a heterogeneous structure (Han et al., 2020) and laboratory-scale research previously reported the distributions of microbial communities among different sampling sections of the anaerobic reactor (Ambuchi et al., 2016), no obvious clustering patterns existed among different layers of the EGSB tank in this study.

Further, we found that the changing physicochemical properties had significant associations with the variations of microbial communities; especially the COD, VSS/TSS ratio, and temperature were predominant factors in shaping community dissimilarities among months (Figures 2D,E). Specifically, microbial diversity (Chao1 and Shannon) was significantly higher in winter (December, January, and February) and autumn (September, October, and November; Figure 2B; Table 2). Most months from winter and autumn had higher biogas production, COD removal efficiency, and nitrogen removal efficiency (Table 1; Figure 1), which were accompanied by relatively higher OLR, higher VSS/TSS ratio, and lower temperature. The result was similar to the previous report (Maleki et al., 2018). GBM analysis revealed the major influences of OLR on Chao1 and PD (Supplementary Figure S2), especially in samples from winter determined by RDA analysis (Figure 2F), while the organic loading rate (OLR) is deemed to play a pivotal role in the removal of nutrients from wastewater (Al Ali et al., 2020). Further increased OLR showed positive effects on microbial diversity of the EGSB reactor (Huang et al., 2019; Zhao et al., 2019), and the biodegradable organics could stimulate the proliferation of heterotrophic bacteria under anoxic conditions, which would degrade the toxic organic compounds and improve the nitrogen removal efficiency in the long-term operation of wastewater reactors (Li et al., 2020).

Besides, by taxonomic classification of ASVs, Proteobacteria, Firmicutes, and Bacteroidetes were highly abundant phyla across samples in this anaerobic-activated sludge, which were reported as the popular dominant phyla in the anaerobic digestion and are responsible for complex organic matter degradation and fermentation (Resende et al., 2016; Zhao et al., 2019), providing favorable substrates for methanogenic processes (Pu et al., 2022). The formation of microbial communities in this system was highly diverse in different months. Specifically, Firmicutes and the less abundant Spirochaetes occupied larger proportions in August and April compared with the other months, respectively (Figure 2C). Nevertheless, the species richness (Chao1) was similar in April and August, while a dramatic reduction of Shannon happened in April (Figure 2B). The microbial communities of samples in April seemed to be more influenced by the maldistribution of the dominant phyla than in August. Moreover, the result of RDA indicated that temperature was the major factor in shaping the microbial community structure of samples from August (owning the highest temperature: 39°C), while pH significantly contributed to the formation of microbial composition in April (owning the highest pH value: 7.4; Figure 2F). By the previous research, higher temperatures caused the transition from Bacteroidetes/Proteobacteria to Firmicutes (Hupfauf et al., 2018), while Spirochaetes seemed to prefer weakly basic conditions (Lee et al., 2013).

It is noteworthy that nitrogen removal underlying the anaerobic system is important for wastewater treatment, especially metabolic pathways with energy efficiency and environmentally friendly. The traditional biological reactive nitrogen treatment processes including the DNRA and denitrification primarily appeared in this anaerobic digestion system, which were two competing microbial nitrate-reduction processes. Previous research suggested that the DNRA bacteria generally appeared in anoxia and electron donor-rich environments (Van Den Berg et al., 2015), while the denitrifying bacteria often showed stronger competition for substrates in activated sludge than them (Wang et al., 2020b). Studies reported the spontaneous enrichment of anammox bacteria in the engineered systems and the frequent co-existing of the DNRA and anammox pathways during the anaerobic degradation (Han et al., 2020; Sui et al., 2021; Meng et al., 2023), however, the key genes underlying this ammonium consumption pathway were completely absent in our dataset (Table 3). It might be due to a shortage of anammox inoculum or inhibitory effects in activated sludge on the ANAMMOX process (e.g., substrate, pH, and temperature; Jianlong and Jing, 2005).

The enriched DNRA pathway in EGSB was mainly contributed by the highly abundant key genes involved in it, including *narGH*, *nrfABCDH*, and *nirBD*, which mainly originated from Proteobacteria, Chloroflexi, Nitrospirae, and Bacteroidetes (Figures 3A,B). Previous research suggested that the same functional groups may occupy different niches with different combinations of functional enzymes (Lee and Francis, 2017). We observed that the *narGH* and *nrfABCDH* involved in the DNRA pathway were significantly lower in April (Figures 3C,D), whereas the *nirBD* coupled to *narGHIJ* in DNRA (Stolz and Basu, 2002) were significantly higher in April (Supplementary Figure S3). Moreover, the species with similar genomes (functional capacities) will tend to occupy the same ecological niche, exist in substrate competition, and hence compete with each other (Aristide and Morlon, 2019). In the present study, compared with the highly enriched DNRA pathway, the abundances of most genes participating in assimilatory nitrate reduction pathways were extremely low.

The gene *hcp* was ranked the top three highly abundant genes in this EGSB, which is commonly deemed to participate in hydroxylamine reduction (Tu et al., 2019). However, the abundances of the key genes (*amoABC* and *hao*) in the nitrification pathway were extremely low (Table 3), which resulted in less formation of the substrate hydroxylamine for *hcp*. Interestingly, it is also considered a high-affinity NO reductase participating in the denitrification pathway (Yu et al., 2020), especially under nitrosative (Balasiny et al., 2018) and oxidative stress (Almeida et al., 2006), which meant in this study, the gene *hcp* mainly participated in the denitrification pathway (Supplementary Figure S3).

A significant positive correlation between the total variation of nitrogen-related genes and the physicochemical properties existed among samples (Figure 4A). The concentration of COD and OLR showed the main influence on genes in the DNRA and denitrification pathways (Figure 4B), while previous research pointed out that both DNRA bacteria and denitrifies could use the organic carbon substances from removed COD as electron donors (Zhou et al., 2022).

Having abundant microbial communities, activated sludge is the residence of“microbial dark matters” (MDMs), which play necessary ecological roles but is restricted to being isolated (Zhang and Zhang, 2022). To better determine the composition and nitrogen cycling potential, we conducted metagenome binning and successfully isolated 172 high-quality species genomes covering the reported primary phyla (e.g., Proteobacteria, Planctomycetes, and Bacteroidetes; Supplementary Figure S4). Consistent with gene annotation, the number of MAGs functioning in the DNRA was more than in the assimilatory nitrate reduction pathway, while the MAGs were mainly classified into six phyla (Figure 5). The phylum Planctomycetota were significantly higher in the colder month (spring and winter), while Acidobacteria showed the opposite trend. Two DNRA bacteria belonging to Nitrospirae were significantly higher in autumn. Ali et al. previously reported that in the wastewater treatment plants, populations from the Planctomycetes and Bacteroidetes were enriched during the colder, while Chloroflexi and Nitrospirae had enriched during the warmer months (Al Ali et al., 2020). We found that six denitrifying bacteria recovered in this EGSB all belonged to Proteobacteria (Supplementary Figure S5), which was the predominant denitrifier in different types of wastewater treatment plants (Heylen et al., 2006). Meanwhile, MAG126 (*Pinisolibacter* sp.) was significantly lower in summer, while MAG147 (*Rhizobiales* bacterium) was significantly higher in colder months. A previous study also observed that the *Rhizobiales* were especially enriched in cold seasons (Shi et al., 2022b) indicating they might be psychrophilic bacteria. In addition, dinitrogen can be converted to ammonia by nitrogen fixation microbes in activated sludge (Rose et al., 2021), while we found 26 MAGs were diazotrophs with *anfG* or *nifDHK*, in which eight were archaea. No obvious distribution patterns of each phylum were found among seasons (Supplementary Figure S6).

The majority of underexplored viruses have been recovered from wastewater treatment systems (Shi et al., 2022a), which could affect functional microbiota in nutrient removal and element cycle (Chen et al., 2021). We obtained 3,360 non-redundant putative viral sequences from the anaerobic activated sludge and found the *Siphoviridae*, *Podoviridae*, and *Myoviridae* were the three main viral families, which was consistent with the previous studies (Jankowski et al., 2022; Lin et al., 2022). Great diversity and novelty were observed in viral communities, while their variation among different seasons showed a similar pattern to the microbial communities (Figure 6). Wang et al. also observed the viral regular variations among seasons in wastewater treatment plants (Wang et al., 2020a). The previous study suggested the far more important roles of viruses in the dynamics of the engineered system (Brown et al., 2019). In this study, we confirmed the strongly significant associations between the abundance of recovered populations and viral communities among months, meanwhile, the significant correlations of specific functional members with viral compositions between the colder and warmer months were observed (Figure 7A). However, the viruses and hosts underlying the host-virus linkages were mostly unidentified (Figure 7B), which may lead to the extremely complex and difficult phage-bacterial host system prediction in activated sludge (Du et al., 2021).

## 5. Conclusion

In summary, our study demonstrated that the environmental factors and operation parameters including ambient temperature, COD, OLR, and VSS/TSS ratio mainly contributed to the monthly and seasonal variation of total microbial communities in composition and function. The higher alpha diversity combined with higher VSS/TSS ratio and OLR in relatively low temperatures often leads to higher biogas production and nitrogen removal efficiency. DNRA and denitrification pathways were prime nitrogen removal pathways in this dataset, which were mainly exerted by Proteobacteria, Planctomycetota, and Nitrospirae with great novelty. Besides, the great diversity and novelty of viral communities were also detected in EGSB, which had similar monthly and seasonal abundant variation. Further research should focus on the active populations and active nitrogen cycling genes to better depict the real interactions during changing properties. Meanwhile, the absence of an energy-conserving anammox pathway in the present study should be considered in the future for optimization of the EGSB system.

## Data availability statement

The amplicon sequences and the metagenome sequences were deposited at the NCBI Sequence Read Archive (SRA) under Bioproject Accession No.: PRJNA896246.

## Author contributions

KZ, YZ, and PeW carried out the molecular experiments, analyzed the data, and wrote the manuscript. PaW, MD, and XY carried out data analyses. PeW and XY contributed to the experiments and collected the samples. YZ and WL conceived the study, contributed to the design, and interpreted the research. All authors contributed to the article and approved the submitted version.

## Funding

This work was supported by the Natural Science Foundation of China (No. 51808157), the Natural Science Foundation of Guangdong Province (No. 2018A0303130014), Guangzhou Municipal Science and Technology Project (No. 202002030374), the Innovation Project of Guangdong Colleges and Universities (Nos. 2018GKTSCX091 and 2019GKTSCX012), and the Science and Technology Project of GDITC (Nos KYRC2020-004, KJ2020-003, and CXCYDSGZS-008).

## Conflict of interest

PW was employed by China National Electric Apparatus Research Institute Co., Ltd.

The remaining authors declare that the research was conducted in the absence of any commercial or financial relationships that could be construed as a potential conflict of interest.

## Publisher’s note

All claims expressed in this article are solely those of the authors and do not necessarily represent those of their affiliated organizations, or those of the publisher, the editors and the reviewers. Any product that may be evaluated in this article, or claim that may be made by its manufacturer, is not guaranteed or endorsed by the publisher.

## Supplementary material

The Supplementary material for this article can be found online at: https://www.frontiersin.org/articles/10.3389/fmicb.2023.1125709/full#supplementary-material

SUPPLEMENTARY TABLE S1 Detailed genomic information of the recovered high-quality MAGs.

SUPPLEMENTARY TABLE S2 The similarity evaluation between each MAG and the reference genomes from the NCBI RefSeq database by FastANI.

SUPPLEMENTARY TABLE S3 The number of nitrogen-related genes in each MAG.

SUPPLEMENTARY TABLE S4 Result of the similarity blasting between viral sequences and the CRISPR-Cas spacers identified from MAGs.

SUPPLEMENTARY TABLE S5 Result of the similarity blasting between viral sequences and MAGs.

SUPPLEMENTARY FIGURE S1 Sampling schematic diagram of the EGSB reactor.

SUPPLEMENTARY FIGURE S2 The relative contributions of different physicochemical properties to the alpha indices (Chao1, Faith_PD, and Shannon) are determined by GBM analysis.

SUPPLEMENTARY FIGURE S3 The relative abundance of the highly abundant genes *hcp*, *nifDHK*, *nirBD*, *nirKS*, *norBC*, and *nosZ* among each month and their taxonomic assignment.

SUPPLEMENTARY FIGURE S4 Phylogenetic placement of 172 recovered MAGs. Different colors represent the primary different phyla.

SUPPLEMENTARY FIGURE S5 Phylogenetic tree of the recovered MAGs in denitrification and significant difference among the four seasons of each MAG. Different lowercase letters indicate significant differences among different seasons (Kruskal-Wallis test, *p* < 0.05).

SUPPLEMENTARY FIGURE S6 Phylogenetic tree of the recovered MAGs in nitrogen fixation and significant difference among the four seasons of each MAG. Different lowercase letters indicate significant differences among different seasons (Kruskal-Wallis test, *p* < 0.05).

SUPPLEMENTARY FIGURE S7 The significant difference in the relative abundance of *Siphoviride*, *Podoviridae*, and *Myoviridae* among seasons. Different lowercase letters indicate significant differences among different seasons (Kruskal-Wallis test, *p* < 0.05).

## Figures and Tables

**Table:** 

EGSB	Expanded granular sludge bed
VSS	Volatile suspended solids
TSS	Total suspended solids
VSS/TSS ratio	The ratio of volatile suspended solids (VSS) to total suspended solids (TSS)
OLR	Organic loading rate
COD	Chemical oxygen demand
HRT	Hydraulic retention time
ASVs	Amplicon sequence variants
GBM	Generalized boosted regression modeling
RDA	Redundancy analysis
PCA	Principle component analysis
MAGs	Metagenome-assembled genomes
AMGs	Auxiliary metabolic genes
AF	Aligned fraction
DNRA	Dissimilatory nitrate reduction to ammonia
Anammox	Anaerobic ammonium oxidation
*hdh*	Hydrazine dehydrogenase gene
*hzs*	Hydrazine synthase gene
*narGH*/*nxrAB*	Nitrate reductase/nitrite oxidoreductase gene
*nrfABCDH*	Nitrite reductase gene
*nifDHK*	Nitrogenase molybdenum-iron gene
*hcp*	Hydroxylamine reductase gene
*nirBD*	Nitrite reductase (NADH) gene
*nirK*/*nirS*	Nitrite reductase (NO-forming) gene
*norBC*	Nitric oxide reductase gene
*nosZ*	Nitrous-oxide reductase gene
*narIJ*	Nitrate reductase molybdenum cofactor assembly chaperone
*napAB*	Nitrate reductase (cytochrome)
*nasAB*	Assimilatory nitrate reductase
*amoABC*	Ammonia monooxygenase
*nirA*	Ferredoxin-nitrite reductase
*hao*	Hydroxylamine dehydrogenase
*AnfG*	Nitrogenase delta subunit
*NR*	Nitrate reductase (NAD(P)H)
*narB*	Ferredoxin-nitrate reductase
*NIT-6*	Nitrite reductase (NAD(P)H)
*terL*	Terminase large subunits

## Footnotes

^1^ https://github.com/qiime2/q2-dada2
