# Supplementary figures and images for: Monthly dynamics of microbial communities and variation of nitrogen-cycling genes in an industrial-scale expanded granular sludge bed reactor
Source: Front Microbiol. 2023 Feb 16;14:1125709. doi: 10.3389/fmicb.2023.1125709 (PMC9978346; doi:10.3389/fmicb.2023.1125709)

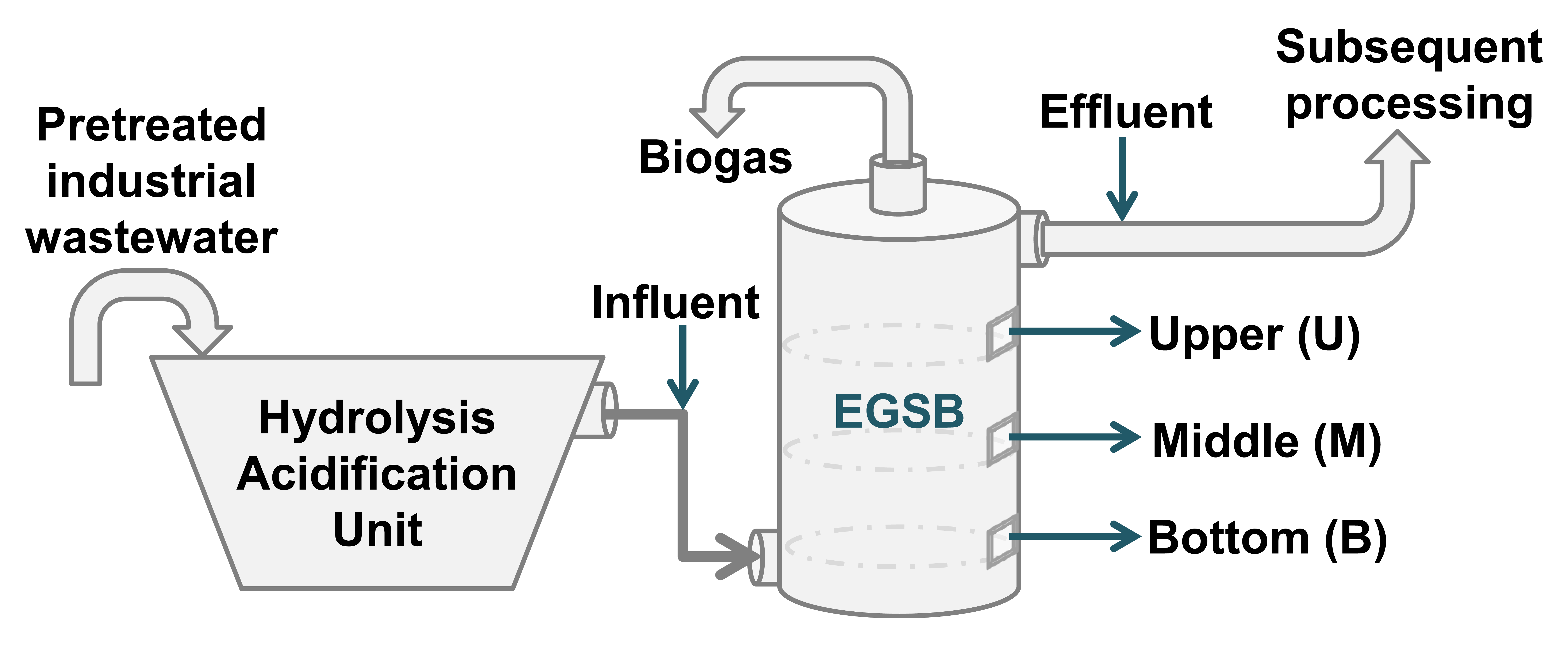

Supplement: SUPPLEMENTARY FIGURE S1 — Sampling schematic diagram of the EGSB reactor. [file Image_1.TIF]

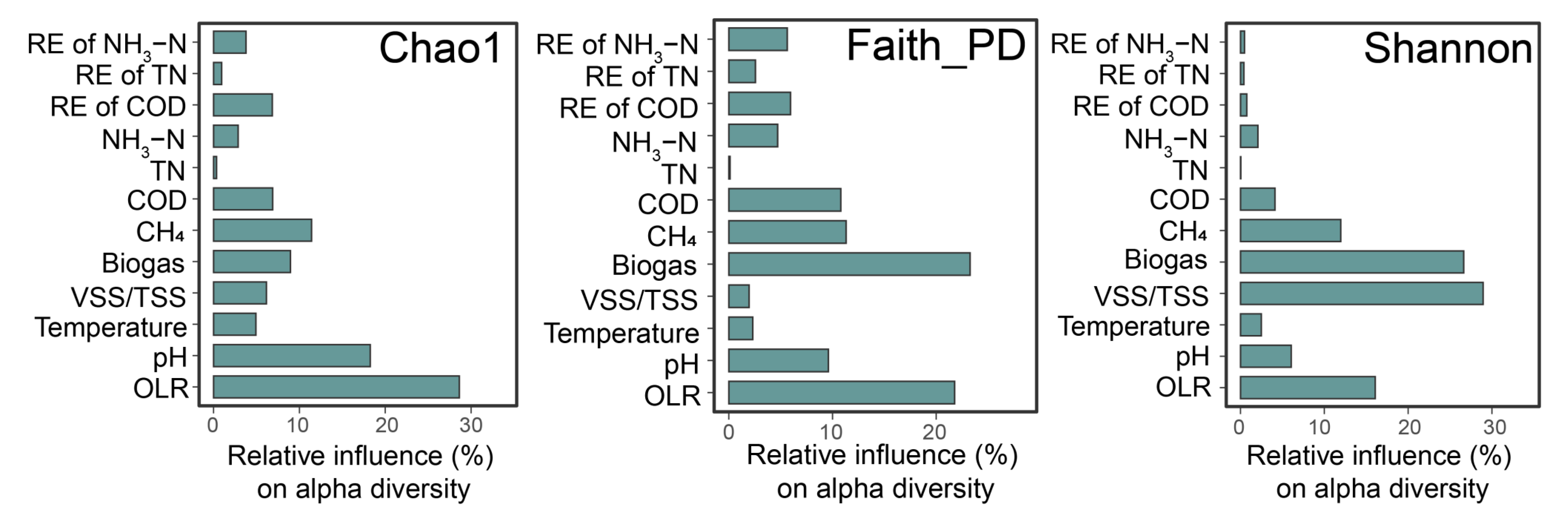

Supplement: SUPPLEMENTARY FIGURE S2 — The relative contributions of different physicochemical properties to the alpha indices (Chao1, Faith_PD, and Shannon) are determined by GBM analysis. [file Image_2.TIF]

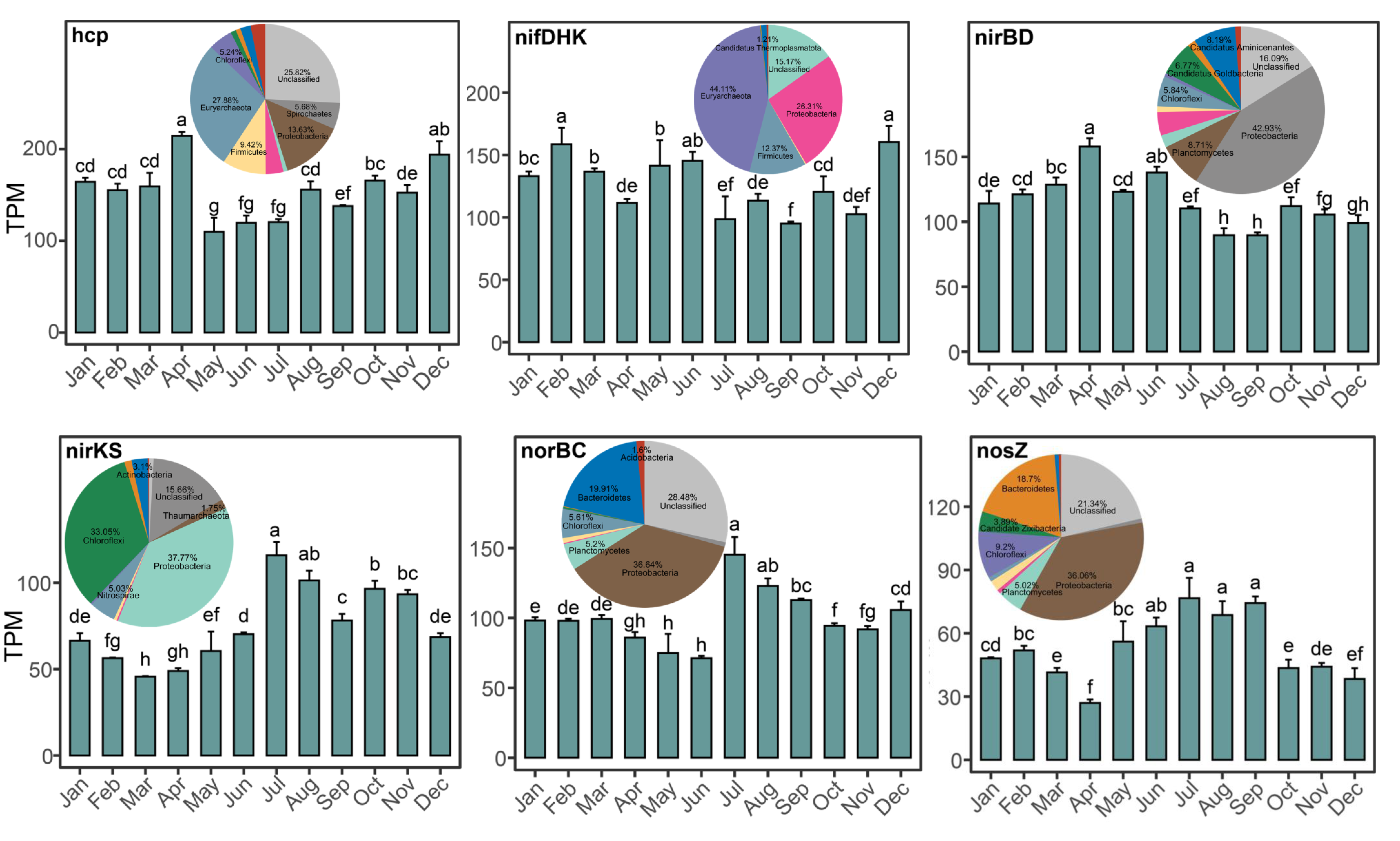

Supplement: SUPPLEMENTARY FIGURE S3 — The relative abundance of the highly abundant genes hcp, nifDHK, nirBD, nirKS, norBC, and nosZ among each month and their taxonomic assignment. [file Image_3.TIF]

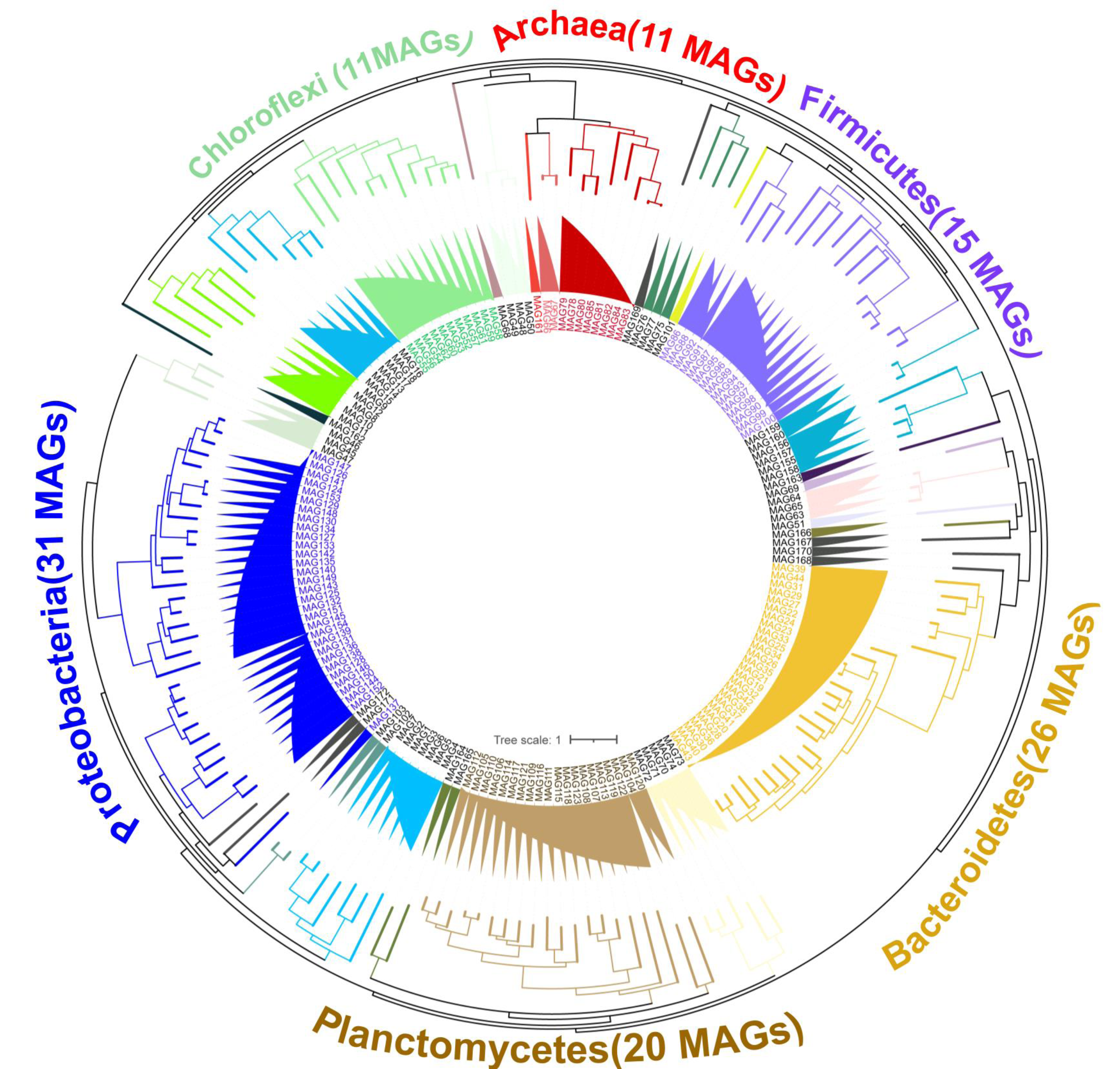

Supplement: SUPPLEMENTARY FIGURE S4 — Phylogenetic placement of 172 recovered MAGs. Different colors represent the primary different phyla. [file Image_4.TIF]

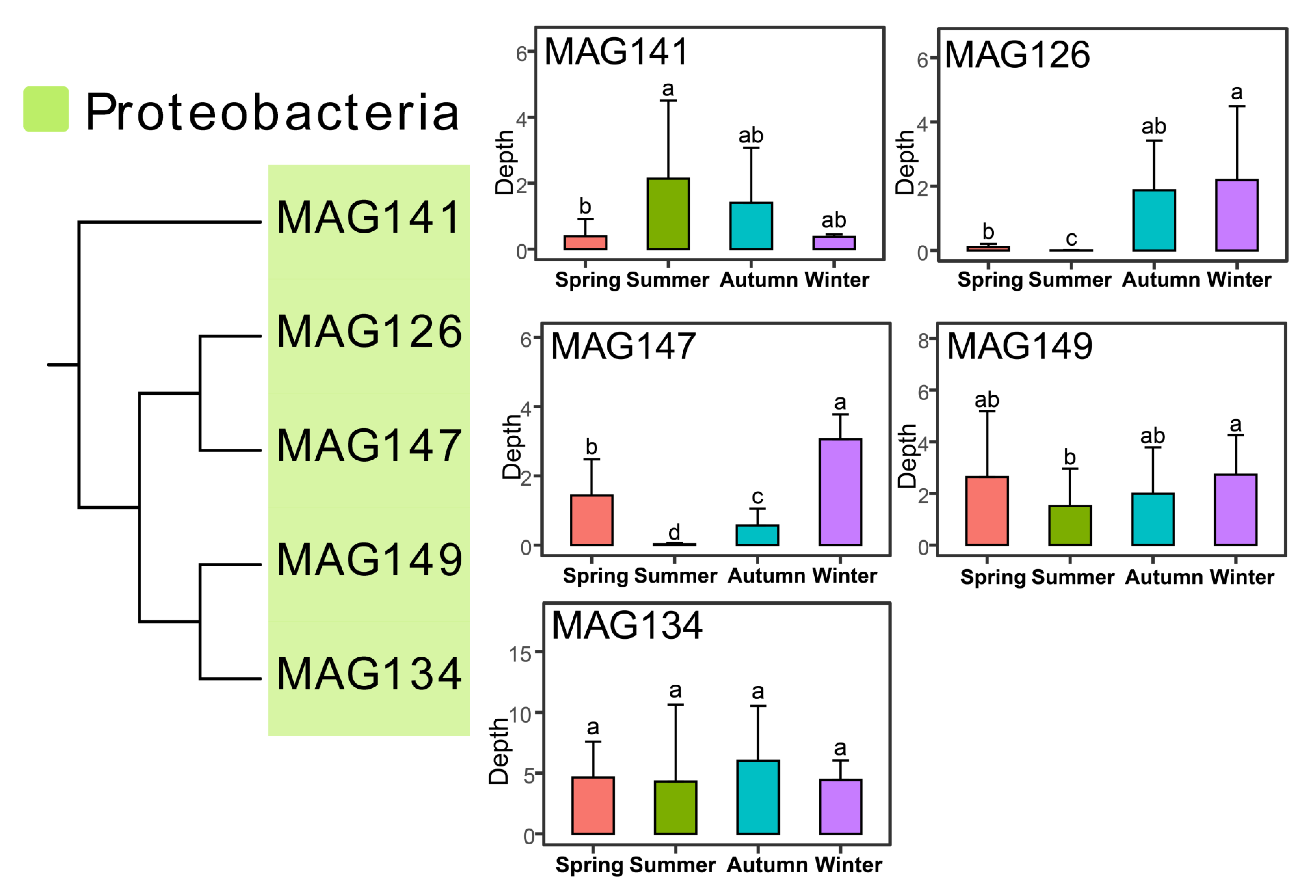

Supplement: SUPPLEMENTARY FIGURE S5 — Phylogenetic tree of the recovered MAGs in denitrification and significant difference among the four seasons of each MAG. Different lowercase letters indicate significant differences among different seasons (Kruskal-Wallis test, p < 0.05). [file Image_5.TIF]

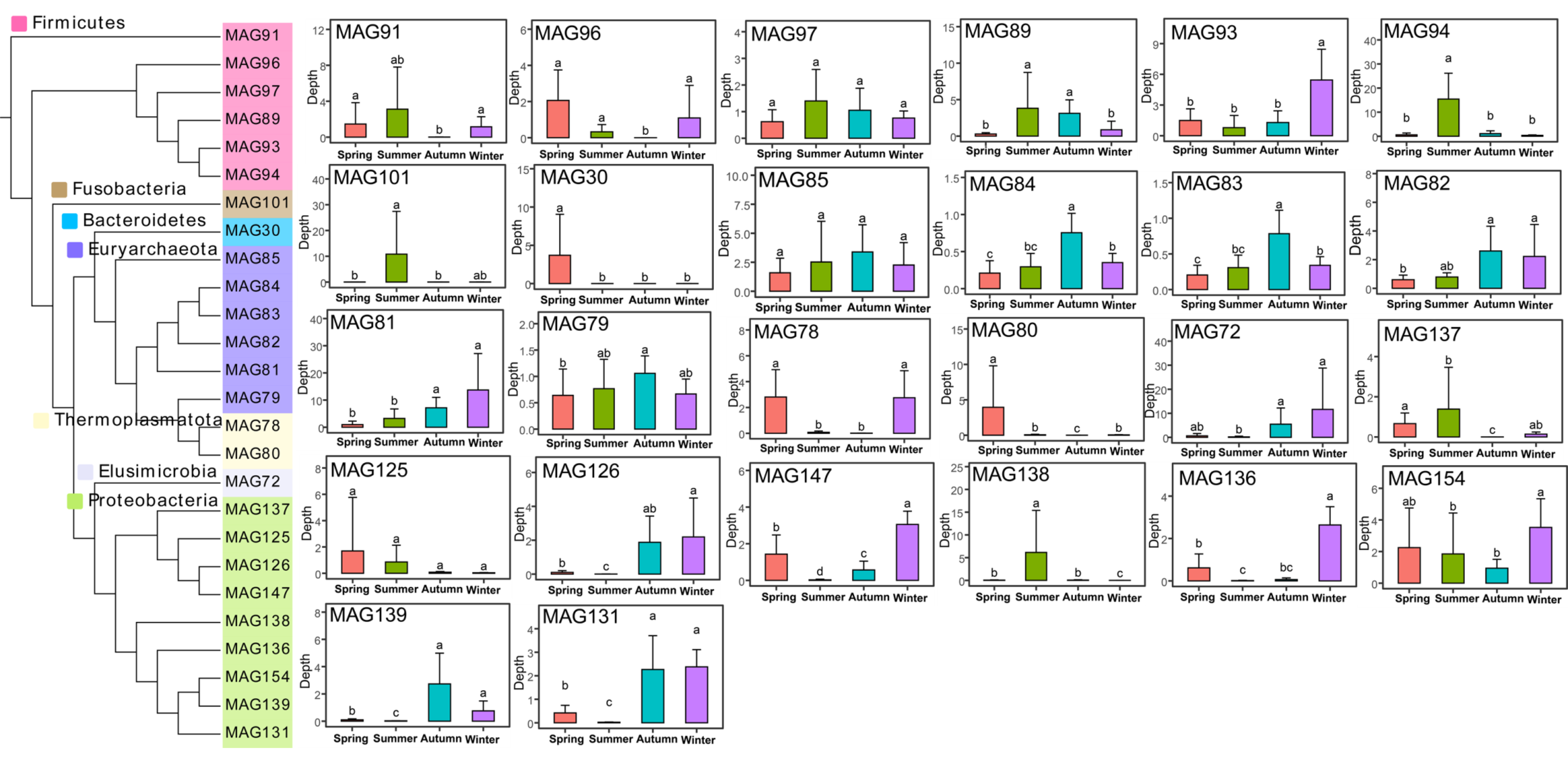

Supplement: SUPPLEMENTARY FIGURE S6 — Phylogenetic tree of the recovered MAGs in nitrogen fixation and significant difference among the four seasons of each MAG. Different lowercase letters indicate significant differences among different seasons (Kruskal-Wallis test, p < 0.05). [file Image_6.TIF]

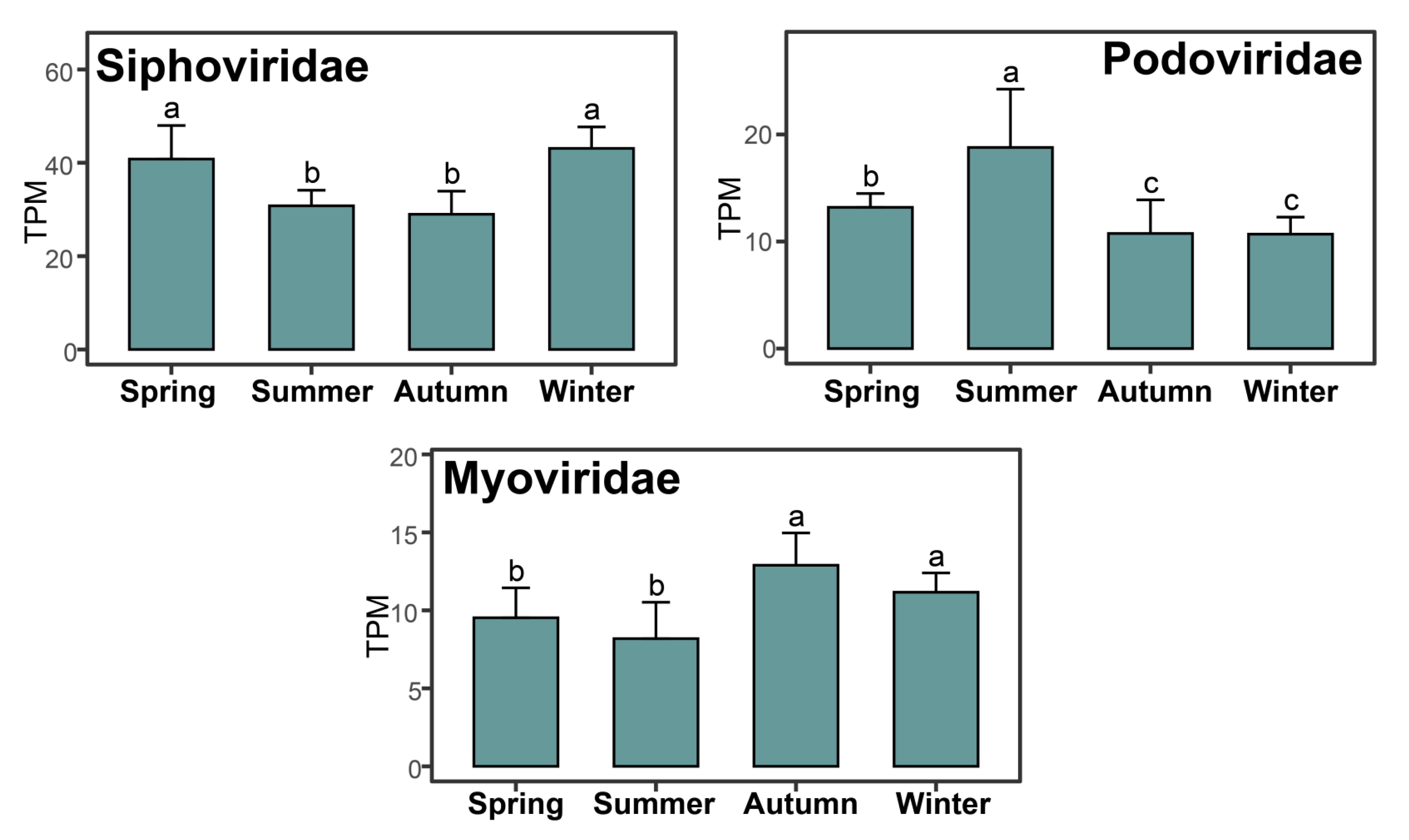

Supplement: SUPPLEMENTARY FIGURE S7 — The significant difference in the relative abundance of Siphoviride, Podoviridae, and Myoviridae among seasons. Different lowercase letters indicate significant differences among different seasons (Kruskal-Wallis test, p < 0.05). [file Image_7.TIF]
